# The safety and feasibility of laparoscopic anatomical left hemihepatectomy along the middle hepatic vein from the head side approach

**DOI:** 10.3389/fonc.2024.1368678

**Published:** 2024-05-24

**Authors:** Wen Li, Lu Fang, Yong Huang

**Affiliations:** Department of General Surgery, The Second Affiliated Hospital, Jiangxi Medical College, Nanchang University, Nanchang, China

**Keywords:** laparoscopic, hepatectomy, left hemihepatectomy, hepatolithiasis, survival

## Abstract

**Background:**

Laparoscopic left hemihepatectomy (LLH) is commonly used for benign and malignant left liver lesions. We compared the benefits and drawbacks of LLH from the head side approach (LLHH) with those of conventional laparoscopic left hemihepatectomy (CLLH). This study was conducted to investigate the safety and feasibility of LLHH by comparing it with CLLH.

**Methods:**

In this study, 94 patients with tumor or hepatolithiasis who underwent LLHH (*n* = 39) and CLLH (*n* = 55) between January 2016 and January 2023 were included. The preoperative features, intraoperative details, and postoperative outcomes were compared between the two groups.

**Results:**

For hepatolithiasis, patients who underwent LLHH exhibited shorter operative time (*p* = 0.035) and less blood loss (*p* = 0.023) than those who underwent CLLH. However, for tumors, patients undergoing LLHH only showed shorter operative time (*p* = 0.046) than those undergoing CLLH. Moreover, no statistically significant differences in hospital stay, transfusion, hospital expenses, postoperative white blood cell (WBC) count, alanine aminotransferase (ALT), and aspartate aminotransferase (AST) were observed between the two groups (*p* > 0.05) for tumor or hepatolithiasis. For hepatocellular carcinoma (HCC), no differences in both overall survival (*p* = 0.532) and disease-free survival (*p* = 0.274) were observed between the two groups.

**Conclusion:**

LLHH is a safe and feasible surgical procedure for tumors or hepatolithiasis of the left liver.

## Introduction

Owing to the rapid development of the laparoscopic field, laparoscopic hepatectomy has been proven to be safe and reliable (1–11). Laparoscopic left hemihepatectomy (LLH) is particularly suitable because of the unique structure of the left liver lobe: it can be easily exposed, it occupies a smaller volume within the abdominal cavity, it has a relatively independent and acute angle tract, and it exhibits clear vasculature gradation (7, 12). LLH is routinely and widely employed for the treatment of benign and malignant lesions of the left liver.

In conventional left hemihepatectomy, the liver parenchyma is transected from the ventral side toward the root along the trunk of the middle hepatic vein (MHV) (13). As the MHV is located in the deep part of the liver parenchyma, the liver tissue must be split more ventrally to determine the landmark. Simultaneously, finding the main trunk from the branch of the MHV can easily pull and tear the tube wall, leading to venous bleeding. Recently, scholars have proposed that LLH from the head side approach can avoid these shortcomings (14–16). Thus, by simply separating along the plane of the umbilical vein ligament and slitting a small section of the liver parenchyma on the back side, the main trunk of the MHV can be found. The “head side approach” is used to disconnect the head of the liver parenchyma from the tail along the foot side of the main hepatic vein. Thus, ligating the affected collateral branches separately reduces the risk of hepatic vein tearing.

To date, there have only been a few reports on comprehensive analysis directly comparing the benefits and drawbacks of conventional laparoscopic anatomical left hemihepatectomy (CLLH) with those of the laparoscopic anatomical left hemihepatectomy from the head side approach (LLHH). Therefore, we investigated safety and feasibility by comparing CLLH with LLHH.

## Materials and methods

### Patients and grouping

This clinical study was conducted at the Department of General Surgery and was approved by the Institutional Review Board of the Second Affiliated Hospital of Nanchang University. Informed consent was obtained from all participants. This study was conducted according to established national and institutional ethical guidelines regarding the involvement of human subjects and the use of human tissues for research. In this study, 94 patients with tumor (*n* = 41) or hepatolithiasis (*n* = 53) who underwent LLHH (*n* = 39) and CLLH (*n* = 55) between January 2016 and January 2023 were enrolled. The clinical characteristics of the patients are presented in **Table 1**.

**Table 1:** Characteristics of tumor patients.

	LLHH (*n* = 18)	CLLH (*n* = 23)	*p*
Gender (M/F)	11/7	15/8	0.786
Age (years)	54 ± 12	53 ± 12	0.868
BMI (kg/m^2^)	23.2 ± 3.6	24.3 ± 4.2	0.387
HCC	17	19	0.155
Cirrhosis	13	17	0.903
Child–Pugh (A/B)	15/3	19/4	0.951
Lesion diameter (cm)	6.1 ± 2.7	5.7 ± 3.8	0.255
Margin (mm)	22 ± 13	24 ± 17	0.361
Operative time (min)	151 ± 40	194 ± 77	**0.046**
Blood loss (mL)	179 ± 93	212 ± 117	0.293
Transfusion	2	4	0.577
Hospital stay (days)	8.8 ± 2.6	9.7 ± 2.7	0.290
Hospital expenses (WanRMB)	5.2 ± 1.1	5.6 ± 1.3	0.219
Bile leakage	1	5	0.151
Clavien–Dindo grade	9	15	0.326
I–II	7	11	0.567
III–IV	2	4	0.577
Postoperative laboratory data			
WBC (10^9^/L)	11.2 ± 2.9	12.3 ± 3.2	0.280
AST (U/L)	192 (123–241)	172 (85–250)	0.793
ALT (U/L)	206 (150–247)	137 (105–264)	0.293

LLHH, laparoscopic left hemihepatectomy of the head; CLLH, conventional laparoscopic left hemihepatectomy; HCC, hepatocellular carcinoma; WBC, blood cell count; AST, aspartate aminotransferase; ALT, alanine aminotransferase; WanRMB, ten thousand renminbi.

Bold font indicates statistical significance.

### Surgical procedure

**Figure 1:**
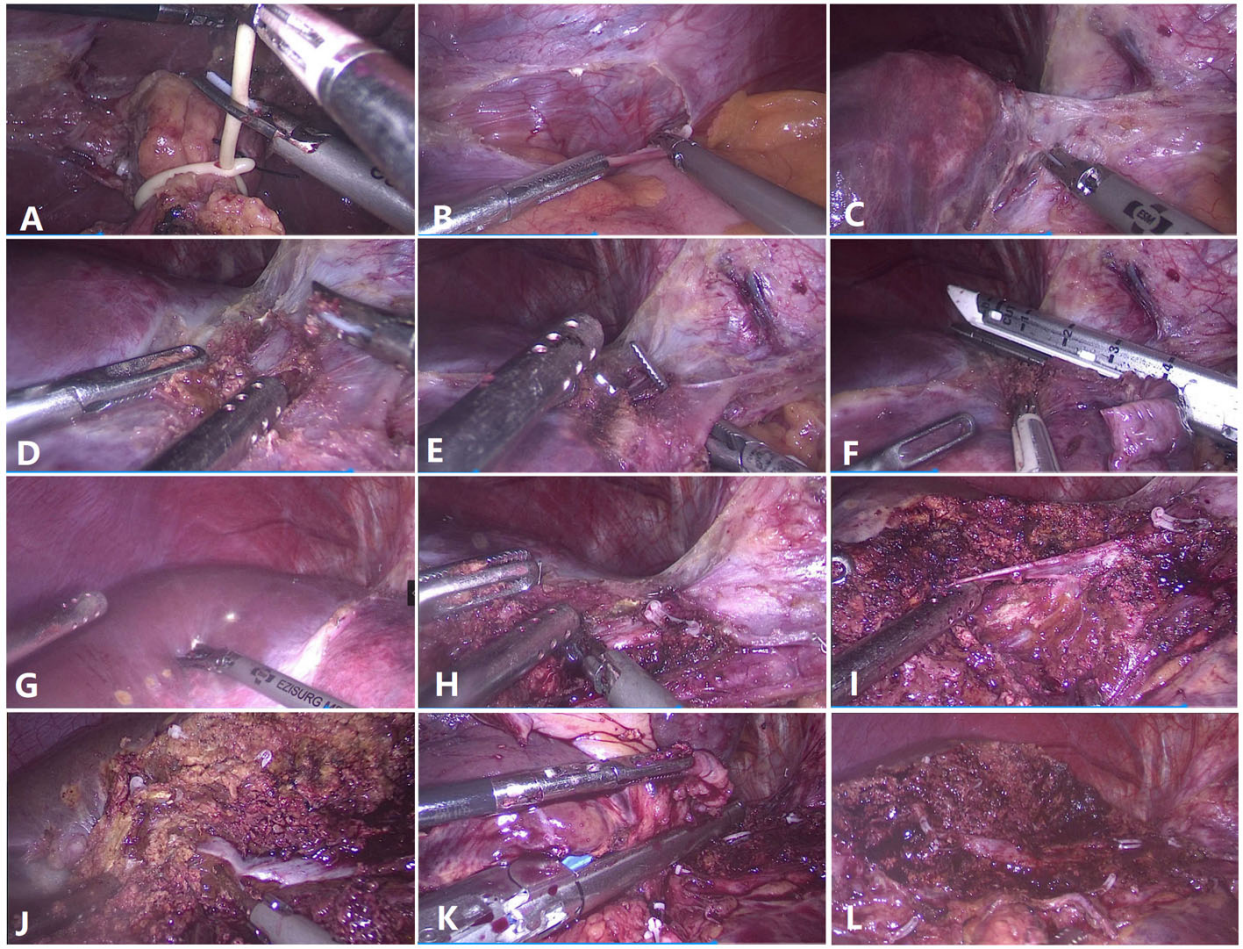
Surgical techniques for laparoscopic anatomical left hemihepatectomy from the head side approach. **(A)** Preset hepatic portal block strap. **(B)** Detachment of the left triangular ligament. **(C)** The left hepatic vein and the Arantius ligament were exposed. **(D)** The root of the left hepatic vein and the middle hepatic vein (MHV) were exposed. **(E)** The left hepatic vein was dissected and detached. **(F)** The left hepatic vein was severed at the root. **(G)** Hepatic ischemic line of the left liver. **(H)** The root of the MHV was exposed. **(I)** The branch (umbilicus fissure vein) of the MHV was dissected and severed. **(J)** Parenchymal transection was performed along the MHV toward the tip direction. **(K)** The left Glissonean pedicle was dissected. **(L)** Liver section after left hemihepatectomy.

**Figure 2:**
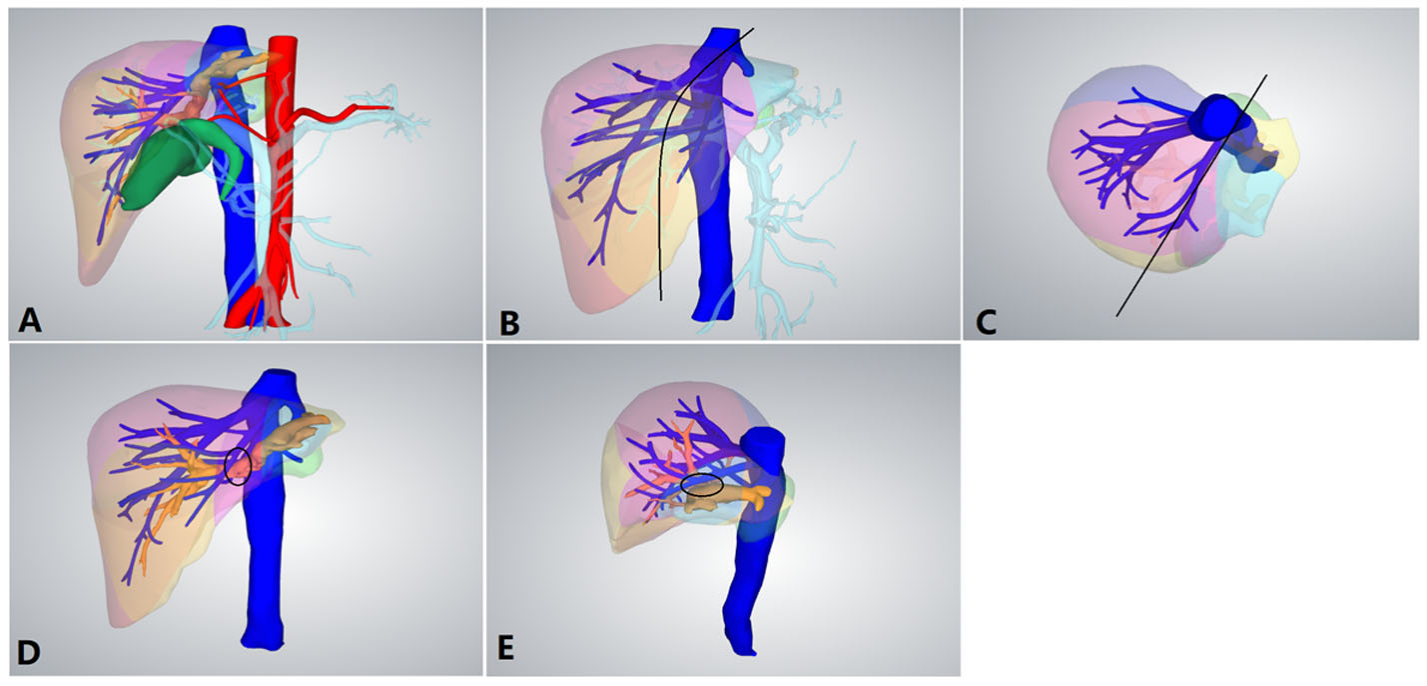
Preoperative 3D reconstruction. **(A)** Preoperative 3D reconstruction. **(B)** Positive view of the expected tangent line (*black line*) of left hemihepatectomy. **(C)** Upper view of the expected tangent line (*black line*) of left hemihepatectomy. **(D)** The left hepatic duct was closely connected to the middle hepatic vein (MHV; *black circle*). **(E)** Side view of the left hepatic duct and MHV.

**Figure 3:**
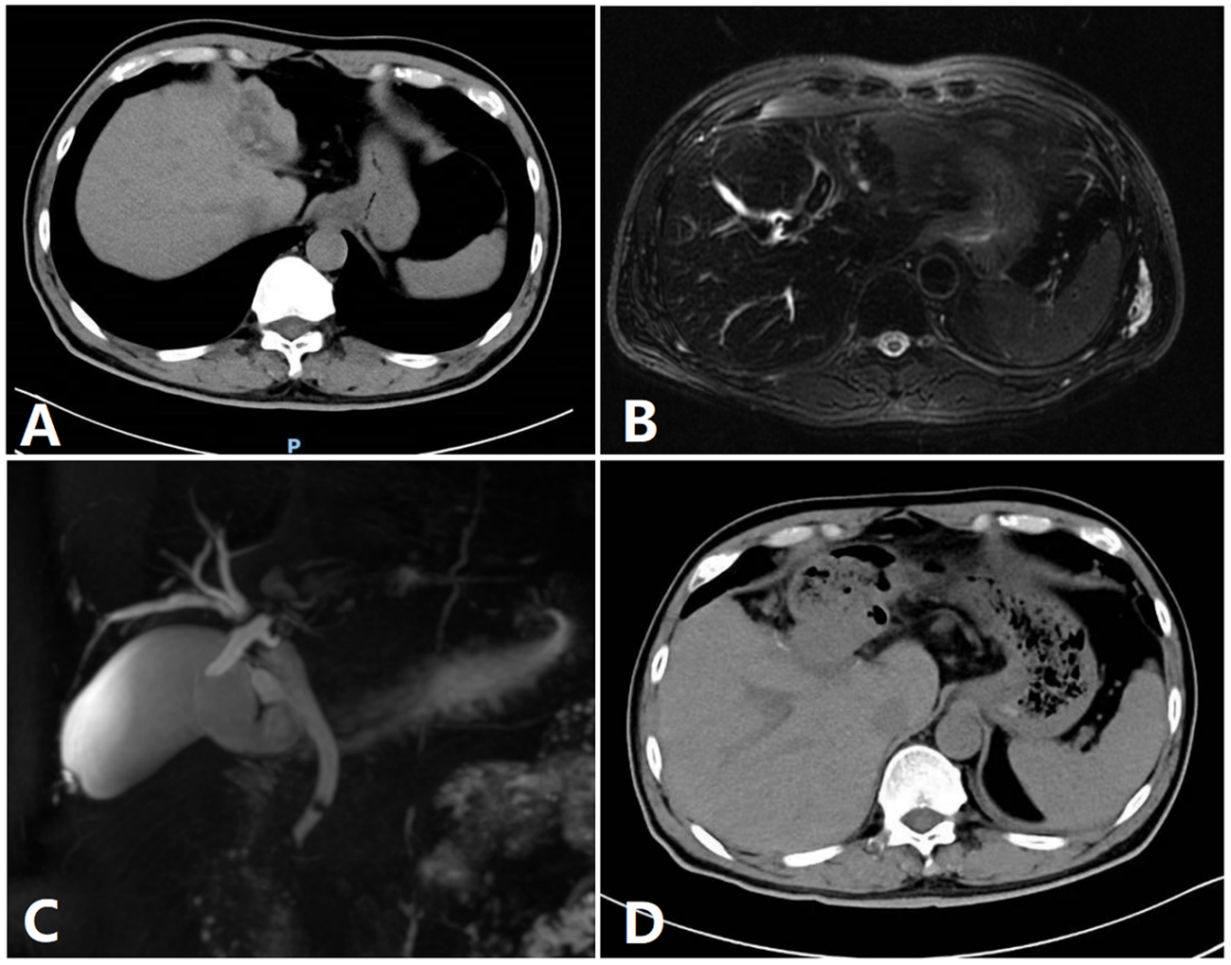
Image of a patient. **(A)** A preoperative CT image of a patient. **(B)** Preoperative MRI. **(C)** Preoperative bile duct water imaging. **(D)** CT imaging after 1 week.

The patients were placed in the semilateral decubitus position without flexing the operative table. Trocars were arranged according to our previous study (8, 9). The specific surgical procedure is depicted in **Figure 1**. The left hemiliver was mobilized. Furthermore, the round and falciform ligaments, the left triangular ligament, and the coronary ligament were severed. Subsequently, the plane of the umbilical vein ligament was separated (Arantius tube), and a small section of the liver parenchyma on the back side was opened to expose the root of the MHV, the left hepatic vein (LHV), and the crypt between them. Evaluation of the preoperative 3D reconstruction of the relationship between the lesions and MHV is presented in **Figure 2**. The LHV was ligated using Hem-o-Lok clips. The “head side approach” was used to disconnect the liver parenchyma from the head to the tail along the foot side of the MHV. Ligating the affected collateral branches separately reduces the risk of hepatic vein tearing. Finally, the left hepatic pedicle was severed using an Endo-GIA stapler. A computed tomography image of a patient is shown in **Figure 3**.

The surgical procedure for CLLH was as follows. Firstly, the ligaments of the left liver were severed. Subsequently, the left hepatic artery and the left portal vein were dissected and severed using an Endo-GIA stapler. The liver parenchyma was transected based on the hepatic ischemic line. The tip of the MHV should be located first before looking for the trunk. The intrahepatic vascular or bile duct was ligated using Hem-o-Lok clips, and then the LHV was disconnected using an Endo-GIA stapler.

### Statistical analysis

Statistical analysis was performed using Statistical Package for the Social Sciences, version 21.0 (IBM Corp., Armonk, NY, USA). Continuous variables are expressed as the mean ± standard deviations or medians (ranges), while categorical variables are expressed as frequencies. Continuous variables were compared using Student’s *t*-test or the Mann–Whitney *U*-test, while categorical variables were compared using the chi-square test or Fisher’s exact test. Results with a *p*-value <0.05 were considered statistically significant.

## Results

### Perioperative outcomes for tumor

A total of 41 patients with tumors who underwent LLHH (*n* = 18) and CLLH (*n* = 23) between January 2016 and January 2023 were enrolled. The preoperative features, intraoperative details, and postoperative outcomes are presented in **Table 1**. No significant intergroup differences in sex, age, body mass index (BMI), lesion diameter, or liver function were noted (*p* > 0.05).

Patients who underwent LLHH had a shorter operative time (151 ± 40 min *vs.* 194 ± 77 min, *p* = 0.046) than those who underwent CLLH. Furthermore, there were no significant differences in blood loss (179 ± 93 mL *vs.* 212 ± 117 mL, *p* = 0.293), transfusion (*p* = 0.577), hospital stay (8.8 ± 2.6 days *vs.* 9.7 ± 2.7 days, *p* = 0.217), hospital expenses [5.2 ± 1.1 WanRMB (ten thousand renminbi) *vs.* 5.6 ± 1.3 WanRMB, *p* = 0.219], or surgical margin (22 ± 13 mm *vs.* 24 ± 17 mm, *p* = 0.361) between the LLHH and CLLH groups. Moreover, no significant differences in the rate of bile leakage (*p* = 0.151), complications (*p* = 0.326), postoperative white blood cell (WBC) count (11.2 ± 2.9 × 10^9^/L *vs.* 12.3 ± 3.2 × 10^9^/L, *p* = 0.280), aspartate aminotransferase (AST) level [192 (123–241) U/L *vs.* 172 (85–250) U/L, *p* = 0.793], and alanine aminotransferase (ALT) level [206 (150–247) U/L *vs.* 137 (105–264) U/L, *p* = 0.293] were observed between the two groups. No patients died in either group.

### Perioperative outcomes for hepatolithiasis

A total of 53 patients with hepatolithiasis who underwent LLHH (*n* = 21) and CLLH (*n* = 32) between January 2016 and January 2023 were enrolled. The preoperative features, intraoperative details, and postoperative outcomes are presented in **Table 2**. No significant differences in sex, age, BMI, presence or absence of common bile duct exploration (CBDE), or liver function (*p* > 0.05) were observed between the LLHH and CLLH groups.

**Table 2:** Characteristics of patients with hepatolithiasis.

	LLHH (*n* = 21)	CLLH (*n* = 32)	*p*
Gender (M/F)	8/5	12/4	0.989
Age (years)	52 ± 13	50 ± 13	0.916
BMI (kg/m^2^)	22.6 ± 3.2	23.2 ± 3.7	0.374
With CBDE	15	21	0.155
Operative time (min)	180 ± 66	233 ± 98	**0.035**
Blood loss (mL)	221 ± 108	297 ± 121	**0.023**
Transfusion	3	5	0.841
Hospital stay (days)	9.6 ± 3.2	10.6 ± 2.8	0.276
Hospital expenses (WanRMB)	5.5 ± 1.2	5.9 ± 1.1	0.195
Bile leakage	3	10	0.164
Clavien–Dindo grade	15	28	0.147
I–II	12	21	0.537
III–IV	3	7	0.494
Postoperative laboratory data			
WBC (10^9^/L)	12.5 ± 3.5	13.5 ± 3.7	0.335
AST (U/L)	219 (98–258)	238 (122–331)	0.403
ALT (U/L)	218 (140–250)	242 (105–333)	0.501

LLHH, laparoscopic left hemihepatectomy of the head; CLLH, conventional laparoscopic left hemihepatectomy; CBDE, common bile duct exploration; WBC, blood cell count; AST, aspartate aminotransferase; ALT, alanine aminotransferase; WanRMB, ten thousand renminbi.

Bold font indicates statistical significance.

Patients who underwent LLHH had a shorter operative time (180 ± 66 min *vs.* 233 ± 98 min, *p* = 0.035) and less blood loss (221 ± 108 mL *vs.* 297 ± 121 mL, *p* = 0.023) than those who underwent CLLH. No significant differences were noted in transfusion (*p* = 0.841), hospital stay (9.6 ± 3.2 days *vs.* 10.6 ± 2.8 days, *p* = 0.276), and hospital expenses (5.5 ± 1.2 WanRMB *vs.* 5.9 ± 1.1 WanRMB, *p* = 0.195) between the LLHH and CLLH groups. Furthermore, the rate of bile leakage (*p* = 0.151), complications (*p* = 0.326), postoperative WBC counts (11.2 ± 2.9 × 10^9^/L *vs*. 12.3 ± 3.2 × 10^9^/L, *p* = 0.280), and the AST [192 (123–241) U/L *vs*. 172 (85–250) U/L, *p* = 0.793] and ALT levels [206 (150–247) U/L *vs*. 137 (105–264) U/L, *p* = 0.293] were examined between the two groups. No patients died in either group.

### Survival and recurrence for HCC

The survival of patients with hepatocellular carcinoma (HCC) was estimated using Kaplan–Meier survival analysis. The results revealed no significant differences in the overall survival (*p* = 0.532) and disease-free survival (*p* = 0.274) between the LLHH and CLLH groups.

## Discussion

The selection of a reasonable surgical approach is crucial to maximizing patient benefits, particularly in LLH. Moreover, optimization of the surgical program to make the surgery safer and more reliable, subsequently maximizing patient benefits, is our current goal. In this study, we investigated the safety and feasibility of LLHH by comparing it with those of CLLH. The results revealed that LLHH offers several advantages over CLHH, including a shorter operative time and less blood loss.

The results suggest that LLHH is associated with a shorter operative time than CLLH; however, LLHH is associated with less blood loss for patients with hepatolithiasis only. These findings are consistent with those of previous research (16). In our opinion, this is where the advantage of LLHH lies.

1) The trunk of the MHV is located in the deep dorsal side of the liver, and exposing it through CLHH requires the dissection of a large section of the liver parenchyma, which will increase the surgical time and the intraoperative bleeding volume. However, dissection through the dorsal side only requires the dissection of a small section of the liver parenchyma to fully expose it for LLHH.
2) The flow (left hepatic pedicle) and outflow (LHV) tracts of the left liver were controlled during surgery. The MHV and its branches were the only bleeding factors to be considered; therefore, intraoperative bleeding will be reduced.
3) Considering the thicker diameter and wall of the MHV, anatomical left hemihepatectomy through the cephalic approach can safely and effectively expose the MHV for LLHH.
4) The pathway of liver dissection is from the head side to the foot side along the MHV, making it less prone to being lost during liver transection. Particularly for the non-trunk type of the MHV (16), its branches bifurcate in the middle or end in the shape of a “ 人 ,” which increases the difficulty of CLLH and will take more time to process and expose the MHV.

LLHH can effectively overcome the aforementioned difficulties. Moreover, there were more complex anatomical structures caused by cholangitis, hepatic duct dilation, and left liver atrophy for hepatolithiasis, as depicted in **Figure 2** (17). The dilated left hepatic duct is connected to the MHV and must be separated from its wall. During surgery, a considerable amount of time is required to deal with the relationship between the MHV and bile ducts. Furthermore, for patients with hepatolithiasis, the probability of intraoperative bleeding or even damage to the MHV increases significantly. LLLH further demonstrates its advantages. It is particularly suitable for patients with hepatic duct dilation and left liver atrophy. Moreover, LLHH clearly displays the MHV and its branches, and its intraoperative bleeding volume is less than that of CLLH, which is extremely consistent with the actual situation. The advantage of this approach is that it can expose and control the MHV in the proximal area far from the dilated hepatic duct, thereby reducing the risk of bleeding.

Owing to the difficulties of CLLH, surgeons will attempt to avoid the MHV in order to decrease the risk of bleeding when liver parenchyma transection is performed. It is inevitable that some liver tissue will remain, which will lead to bile leakage, postoperative abdominal infection, and even postoperative tumor recurrence. Furthermore, for hepatolithiasis, CLLH has been reported to increase the risk of postoperative complications, such as bile leakage and infection, due to injury to the intrahepatic duct system (14). However, although there were many cases of bile leakage in this study for CLLH, no statistical significance was observed. We believe that there are three main reasons for this: 1) the surgical skill of the surgeons has made significant progress, which was adequate to overcome the difficulties of CLLH; 2) the anesthesiologist effectively controlled the central venous pressure during surgery; and 3) the number of cases may have been insufficient. Moreover, the results revealed that the costs of CLLH were slightly higher than those of LLHH. This is because, in LLHH, the liver parenchyma was transected along the trunk of the MHV from the root toward the tip, with fewer Glisson pipes on the cross-section, which can reduce the use of vascular clamps during surgery (14, 16). Furthermore, the root of the LHV was well exposed, and Hem-o-Lok clips can be used instead of a cutting and closing device, thereby reducing medical costs (18, 19). Our results indicated no significant differences in the complications or the surgical margins between the two groups for tumors. Furthermore, no significant differences in the postoperative WBC counts and the ALT and AST levels were observed, suggesting no significant difference in the extent of perioperative functional outcomes. Moreover, the results suggest that the two surgical methods have no effect on the prognosis of patients with HCC.

From the aforementioned results, it can be noted that LLHH has the following advantages:

1) The main trunk of the MHV can be quickly located using the cephalic dorsal approach, thus significantly reducing the surgical time.
2) The risk of damaging the MHV and its branches is reduced, significantly decreasing the intraoperative bleeding volume and the risk of postoperative hepatic cross-section bleeding.
3) The failure risk of the complete exposure of the MHV in the liver due to bleeding is reduced.
4) Cutting off the liver parenchyma through the MHV via the head back approach can avoid secondary damage to the preserved side of the intrahepatic duct and can reduce the residual inactivated liver tissue adjacent to the MHV, thereby reducing the risk of bile leakage and postoperative infection.
5) Detaching the LHV before segmenting the liver parenchyma reduces the chance of cancer cells entering the blood circulation, which may benefit the long-term prognosis of patients.

In conclusion, LLHH is a safe and feasible surgical procedure for the treatment of tumors or hepatolithiasis of the left liver. In the era of minimally invasive surgical innovation, we hope that this study motivates other centers to improve their surgical methods and benefit patients.

## Data availability statement

The raw data supporting the conclusions of this article will be made available by the authors, without undue reservation.

## Ethics statement

The studies involving humans were approved by the Second Affiliated Hospital of Nanchang University Ethics Committee. The studies were conducted in accordance with the local legislation and institutional requirements. Written informed consent for participation in this study was provided by the participants’ legal guardians/next of kin. Written informed consent was obtained from the individual(s), and minor(s)’ legal guardian/next of kin, for the publication of any potentially identifiable images or data included in this article.

## Author contributions

WL: Writing – original draft, Writing – review & editing, Formal analysis, Investigation, Software, Funding acquisition. LF: Writing – review & editing. YH: Data curation, Formal analysis, Methodology, Software, Writing – original draft, Writing – review & editing, Funding acquisition.
